# Insulator-to-metal-like transition in thin films of a biological metal-organic framework

**DOI:** 10.1038/s41467-023-38434-4

**Published:** 2023-05-19

**Authors:** Pooja Sindhu, K. S. Ananthram, Anil Jain, Kartick Tarafder, Nirmalya Ballav

**Affiliations:** 1 Department of Chemistry, Indian Institute of Science Education and Research, Dr. Homi Bhabha Road, Pune, 411 008 India; 2 Department of Physics, National Institute of Technology Karnataka, Surathkal, Mangalore, 575 025 India; 3 Solid State Physics Division, Bhabha Atomic Research Centre, Mumbai, 400085 India; 4 Homi Bhabha National Institute, Anushakti Nagar, Mumbai, 400094 India

**Keywords:** Metal-organic frameworks, Electronic devices

## Abstract

Temperature-induced insulator-to-metal transitions (IMTs) where the electrical resistivity can be altered by over tens of orders of magnitude are most often accompanied by structural phase transition in the system. Here, we demonstrate an insulator-to-metal-like transition (IMLT) at 333 K in thin films of a biological metal-organic framework (bio-MOF) which was generated upon an extended coordination of the cystine (dimer of amino acid cysteine) ligand with cupric ion (spin-1/2 system) – without appreciable change in the structure. Bio-MOFs are crystalline porous solids and a subclass of conventional MOFs where physiological functionalities of bio-molecular ligands along with the structural diversity can primarily be utilized for various biomedical applications. MOFs are usually electrical insulators (so as our expectation with bio-MOFs) and can be bestowed with reasonable electrical conductivity by the design. This discovery of electronically driven IMLT opens new opportunities for bio-MOFs, to emerge as strongly correlated reticular materials with thin film device functionalities.

## Abstract (web summary)

Temperature-induced insulator-to-metal transitions are usually accompanied by structural phase transitions. Here the authors demonstrate an enhancement of the electrical conductance in a thin film of a biologically relevant metal-organic framework, without noticeable change in the structure, assigned to be of mainly electronic origin.

## Introduction

Controlling charge and spin degrees of freedom in materials by external stimuli like heat, light, electric field, and the magnetic field is technologically demanding. One example is the spin-crossover phenomenon, where two or more distinct spin-states can be reversibly switched, usually varying the temperature of the system^1^. Another example is the resistive switching phenomenon, where the electrical resistance can be reversibly switched between a high-resistance state (insulating) and a low-resistance state (conducting) under the influence of an electric field^2,3^. Resistive switching is at the heart of the operation principles of non-volatile random-access computer memory devices, commonly referred to as memristors^4,5^. Insulators explored so far are mostly transition metal oxides (TMOs), and the switching event can be categorized in general as filamentary or interface type^2,3^. With an elegant demonstration of the electrically addressable memory with modest volatility^6^, reticular materials such as metal–organic frameworks (MOFs)^7,8^ have recently emerged as the second-generation electrical insulators for the development of memristive devices^9^. Various mechanisms like thermochemical, electrochemical, valence-charge (migration as well as re-organization), electrostatics, and ferroelectric polarization were realized with MOFs^6,10–21^; and additional mechanistic pathways are evolving. Specifically, the presence or absence and spatial distribution of guest molecules in the voids of MOF can significantly alter the electrical transport property^22,23^. Therefore, chemical constituents of the MOF and their arrangements (structural) are crucially important in achieving the resistive switching event.

**Fig. 1:**
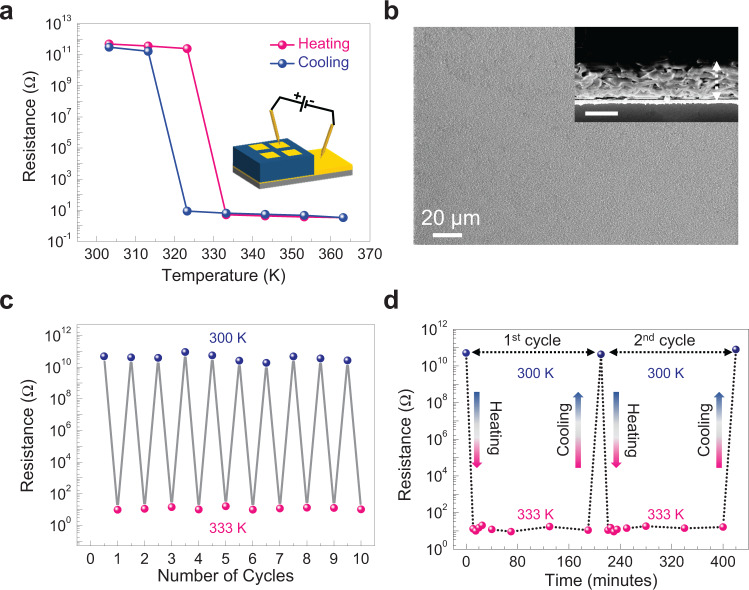
Electrical transport property across the Cu(Cys)_2_ thin film. **a** Temperature-dependent resistance values of the Cu(Cys)_2_ thin film in the cross-plane mode during heating (pink spheres) and cooling (blue spheres) of the sample with a clearly visible hysteretic effect (inset: schematic of the transport measurement with top patterned Au contact and bottom Au substrate as electrodes). Reversible modulation of the resistance values ensured that no diffusion of Au from the contact pads across the Cu(Cys)_2_ thin film took place during the microfabrication process as well during the heating-cooling cycle. **b** Surface morphology of the Cu(Cys)_2_ thin film as revealed by FESEM image (inset: cross-plane FESEM image with a scale bar of 1 μm showing the thickness of the Cu(Cys)_2_ thin film as ~1.2 μm). **c** Cycling stability of the IMLT in the Cu(Cys)_2_ thin film. **d** Time-dependent cycling stability: after measuring the resistance value at 300 K (0 h), the sample was heated to 333 K and kept for a few hours at 333 K; the resistance values were measured at different time intervals, and indeed, no significant change was observed (even by 10 times). Subsequently, the sample was cooled back to 300 K, and the resistance value was measured to be the same as the value at the starting point (0 h). Such behavior was successfully reproduced in the second heating-cooling cycle. Source data are provided as a Source Data file.

The primary advantage of exploring the MOF insulators can be attributed to modular chemical design principles whereby electrical conductivity can be significantly modulated with the choice of metal ion and/or organic ligand as well as filling the pores by redox-active small molecules and/or conducting polymer moieties^24^. The other important factor with MOFs is the fabrication of crystalline thin films on various support substrates by simple step-by-step/layer-by-layer (LbL) technique complementing the ease of control of film thickness^25^. During the last two decades, various biomolecules like nucleobases, peptides, proteins, porphyrins, cyclodextrins, and small biomolecules like amino acids were successfully used as organic building blocks for the generation of various types of biological MOFs^26–28^. It is apparent from the chemical backbones of biomolecules and the predominant ionic bonding scenario that bio-MOFs would be mostly electrical insulators, likewise conventional MOFs. In fact, the electrical transport properties of bio-MOFs are rarely explored, and so is the exploration of resistive switching as well as an insulator-to-metal transition (IMT). Herein, we report thin film (also bulk) synthesis of a bio-MOF, obtained upon reacting the cystine ligand ([SCH_2_CH(NH_2_)CO_2_H]_2_, an oxidized dimer of amino acid cysteine) with Cu(II) ion (spin-1/2), abbreviated here as Cu(Cys)_2_ (Supplementary Figs. 1–6). Just by controlling the temperature, we could reversibly switch the resistance value across the Cu(Cys)_2_ thin film between ~10^11^ Ω at 300 K (insulating state) and ~10 Ω at 333 K (conducting state) at ambient pressure with an additional hysteretic effect (Fig. 1a and Supplementary Figs. 7 and 8)—resembling the recently demonstrated electrical field-induced IMT in thin films of archetypal TMO, vanadium dioxide (VO_2_; spin-1/2), little above room temperature (340 K)^29^.

## Results

As revealed by field-emission scanning electron microscopy (FESEM) images, our ~1.2 μm thick Cu(Cys)_2_ film, fabricated by LbL technique, was uniformly extending over hundreds of microns as well as crystalline (Fig. 1b and Supplementary Fig. 4). Cu(Cys)_2_ thin film was realized to be highly durable under thermal stress whereby the respective resistance values at 300 and 333 K were found to be almost unaltered over ten continued cycles of heating and cooling viz. retention of the “on–off” ratio of ~10^10^ (Fig. 1c). Also, resistance value at 333 K was found to be highly stable over long period of time and the time-dependent stability was successfully reproduced in the subsequent cycles (Fig. 1d). Considering the remarkable change in the resistance value (~10^10^ times), such a thermally-driven phenomenon in the Cu(Cys)_2_ thin film can be assigned to an IMT-like transition (IMLT). Estimated resistivity values for the Cu(Cys)_2_ thin film at 300 and 333 K were found to be ~10^10^ Ωm and ~10^−1^ Ωm, respectively (Supplementary Fig. 7). Further, we have recorded the current–voltage (*I*–*V*) profiles for the Cu(Cys)_2_ thin film from room temperature down to 160 K and no appreciable change in the conductance value was observed, even though the trend was similar to that of an insulator (Supplementary Fig. 9). In case of the IMT involving band-type and Mott-type insulators where the resistivity can be altered over tens of orders of magnitude are most often accompanied by structural phase transition in the system^30^. It is expected that for inherent Mott insulators, IMT, usually induced by temperature, should be of purely electronic origin (without structural change)^31–33^. However, strong Coulomb interaction over bandwidth leads to coupling of charge, spin, and lattice; and eventually, IMT (including the transition to semimetal state^34,35^) involving Mott insulators are also frequently associated with structural change. Notable exceptions of temperature-induced IMT in Mott insulators, without structural change, are surface-tailored layered perovskite Ca_1.9_Sr_0.1_RuO_4_ and compositionally tuned hetero-structured VO_2_ thin film^33,36^.

**Fig. 2:**
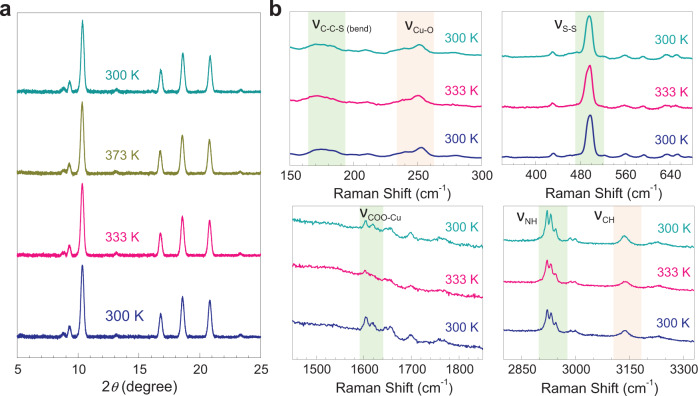
Probing retention of structural integrity by variable temperature XRD and Raman. **a** XRD patterns recorded on the Cu(Cys)_2_ thin film sample at 300, 333, 373, and back to 300 K. The pattern remained almost identical across various temperatures. **b** Raman spectra recorded on the Cu(Cys)_2_ thin film sample at 300, at 333, and back to 300 K. Characteristic vibrational features mentioned and spectral regions are highlighted. Source data are provided as a Source Data file.

We have recorded X-ray diffraction (XRD) patterns on the Cu(Cys)_2_ thin film by systematically varying the temperature from 300 K up to 373 K (and back to 300 K) which could serve as the key marker to investigate any structural change in the system (Fig. 2a and Supplementary Fig. 10). Interestingly, no appreciable change in the XRD patterns could be detected during the heating and cooling cycle. Grazing-incidence XRD (GIXRD) pattern at 300 K ensured that the sharp and separated pattern was not related to any interface effect, but the material itself (Supplementary Fig. 4), and notably, similar XRD patterns were consistently observed in our earlier reports on thin films of coordination polymers. Therefore, in the course of the IMLT, structural order parameters were almost retained in the Cu(Cys)_2_ thin film—primarily an electronic phenomenon, likewise in Ca_1.9_Sr_0.1_RuO_4_ and VO_2_. To complement the temperature-dependent XRD data, we have recorded Raman spectra on the Cu(Cys)_2_ thin film at 300, 333, and back to 300 K (Fig. 2b and Supplementary Fig. 11). Characteristic vibrational signatures were found to be unaltered at different temperatures; specifically, C–C–S bending mode (175 cm^−1^), Cu–O (240 cm^−1^), S–S stretching mode (496 cm^−1^), COO–Cu stretching mode (1603 cm^−1^), C–H (-CH_2_-) stretching modes (2921 cm^−1^, 2932 cm^−1^, and 2945 cm^−1^), and N–H (-NH_2_) stretching mode (3136 cm^−1^)^37,38^. Therefore, Raman spectra confirmed that IMLT in the Cu(Cys)_2_ thin film did not accompany any change in the coordination environment, viz., chemical bonding integrity was retained in the system. One relevant example is the intertwining of redox chemistry and IMT in a MOF induced by oxygen (O_2_) adsorption–desorption chemistry leading to a profound change in the electronic structure throughout the material^39^. Another example is the insulator-to-proton-conductor transition in a dense MOF upon exposure to humidity^40^. Also, solvation-desolvation with water molecules was observed to significantly impact the electrical conductivity of a one-dimensional MOF^41^. However, these examples dealt with a change in the chemical integrity of the systems resembling the chemically-driven IMT in oxides^42^ and unlike the temperature-induced IMLT presented here with a bio-MOF. Notably, in the spectral range of 3000–3800 cm^−1^, no signature of water adsorption-desorption could be detected in our temperature-dependent Raman spectra on the Cu(Cys)_2_ thin film; specifically peaks at 3215 and 3371 cm^−1^ (respective symmetric and asymmetric OH stretch modes of hydrogen-bonded water) as well as a peak at 3683 cm^−1^ (water adsorbed in the pores)^43^ (Supplementary Fig. 11).

**Fig. 3:**
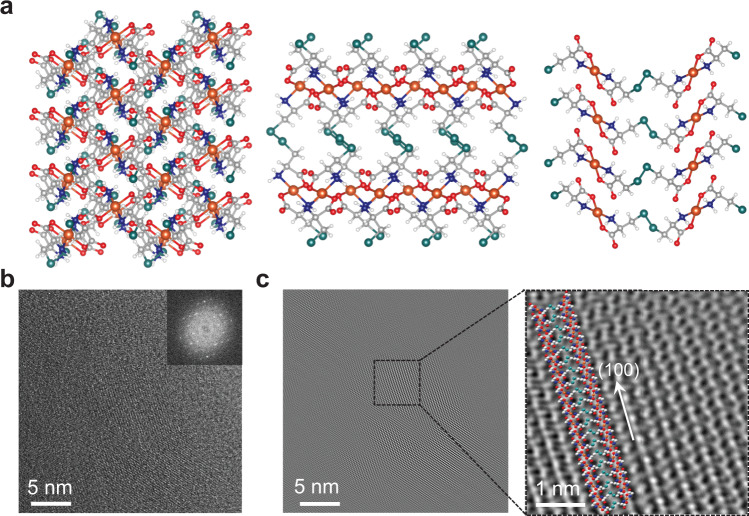
Proposed structure of the Cu(Cys)_2_ system. **a** Structural view from the *a*-axis (left panel), b-axis (middle panel), and *c*-axis (right panel) where carbon, hydrogen, nitrogen, oxygen, sulfur, and copper atoms are represented by gray, white, blue, red, dark cyan and orange colors, respectively. **b** HRTEM image of the Cu(Cys)_2_ sample (inset: corresponding fast-Fourier transform (FFT) image). **c** Inverse fast-Fourier transform (IFFT) pattern extracted from the HRTEM image showing a good overlap with the crystal structure along (100) direction.

One-dimensional (1D) zig–zag chains can be visualized in the simulated crystal structure of our Cu(Cys)_2_ system [Cu(C_3_H_5_NO_2_S)_2_] with four coordination around the Cu(II) ion (two N and two O) and these 1D chains are interconnected by H-bonding giving rise to three-dimensional (3D) framework structure (Fig. 3). Initial geometry of the Cu(Cys)_2_ unit cell was constructed from the reported Zn(Cys)_2_ [Zn(C_3_H_5_NO_2_S)_2_] crystal structure^44^, in which Zn(II) ions were replaced with Cu(II) ions and then the Cu(Cys)_2_ energy-optimised ground state geometry was obtained by performing the density functional theory (DFT) calculations. The energy-optimized structure was subjected to Rietveld refinement of the XRD pattern of the Cu(Cys)_2_ thin film^45^. Preferred orientation was determined by looking at the XRD peak intensities and was applied during the Rietveld refinement^46^, whereby a reasonably fair match between the experimental and calculated XRD patterns was achieved (Supplementary Fig. 12). To complement the XRD data, high-resolution transmission electron microscopy (HRTEM) analysis was performed on an extracted sample from the Cu(Cys)_2_ thin film, and HRTEM image along with the corresponding fast-Fourier transformed (FFT) and inverse fast-Fourier transformed (IFFT) patterns clearly revealed existence of highly periodic 1D zig–zag chains (Fig. 3)^47,48^.

Barring structural phase transition, such a dramatic change in the resistance value in the Cu(Cys)_2_ system can be associated with the contact interface. In earlier work, we have shown thermally driven resistive switching in Cu-TCNQ and Ag-TCNQ (TCNQ = 7,7,8,8-tetracyanoquinodimethane) which was attributed to the alternation of a Schottky barrier at the contact interface, i.e., primarily an interface effect^49^. Specifically, the change in the in-plane resistance value in the Ag-TCNQ thin film at 400 K varied from 10^4^ fold to 10^6^ fold in comparison to the value at 300 K, depending on the type of contact electrode. As for the Cu-TCNQ thin film, the enhancement factor was observed to be 10^4^. Later on, upon increasing the temperature from 300 to 400 K, both in-plane and cross-plane modes, we realized a significant change in the resistance value for the thin film of Cu-TCNQ; and, interestingly, no change in the resistance value for the thin film of Cu-BTC (BTC = 1,3,5-benzenetricarboxylate)^50^. Therefore, it is the intrinsic property of the material, for example, Mott-type (Cu-TCNQ) or band-type (Cu-BTC) insulator^46,51^, which played a more important role than the influence of the interfaces. In the present study, successful capture of the IMLT for the Cu(Cys)_2_ thin film (thickness ~1.2 μm) in both cross-plane and in-plane modes (consistent change in the resistance by 10^10^ fold) clearly endorses that it is not primarily an interface effect. Also, in-plane *I*–*V* profiles on the Cu(Cys)_2_ thin film with various metallic contacts having different work function values (Au, W, and Eutectic-GaIn)^49^ revealed mainly Ohmic-type electrical transport, both at 300 and 333 K; and the change in the resistance by 10^10^ fold was consistent (Supplementary Figs. 13 and 14). Even upon performing in-plane *I*–*V* measurements with patterned Pt pads (having relatively higher work function value of ~6.3 eV) on top of the Cu(Cys)_2_ thin film gave the same results (Supplementary Fig. 15).

**Fig. 4:**
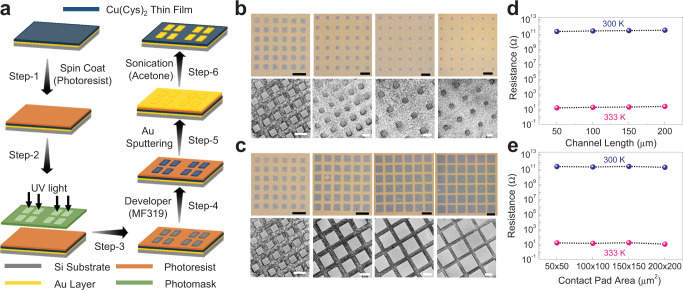
Testing device functionality of the Cu(Cys)_2_ thin film. **a** Schematic of the fabrication of patterned Au pads on top of the Cu(Cys)_2_ thin film by photolithography. **b** Optical (top panels; scale bars represent 200 μm length) and FESEM (bottom panels; scale bars represent 100 μm length) images of the 50, 100, 150, and 200 μm channels (left to right) with Au contact pads on the top of Cu(Cys)_2_ thin film. Scale bars are provided. **c** Optical (top panels; scale bars represent 200 μm length) and FESEM (bottom panels; scale bars represent 100 μm length) images of the 50 × 50 μm^2^, 100 × 100 μm^2^, 150 × 150 μm^2^ and 200 × 200 μm^2^ (left to right) Au contact pads. **d** Plot of resistance values versus and channel length at 300 K (blue spheres) and 333 K (pink spheres). **e** Plot of resistance values versus and contact pad area at 300 K (blue spheres) and 333 K (pink spheres). Source data are provided as a Source Data file.

The robustness of the IMLT phenomenon motivated us to test the device functionality, and accordingly, we have employed photolithography followed by sputtering Au contact pads on the Cu(Cys)_2_ thin film (Fig. 4a). Well-defined patterns with various channel lengths (Fig. 4b) as well as sizes of the Au contact pads (Fig. 4c) can be clearly visualized in the optical and FESEM images. After the microfabrication process involving multi-steps, the structural integrity of the Cu(Cys)_2_ thin film was fully retained, as was reflected in the FESEM images, XRD patterns, and the Raman spectra (Supplementary Fig. 16). The IMLT captured in the cross-plane mode (Fig. 1a) was also successfully captured in our patterned sample in the in-plane mode. Upon systematic variation of the channel length (50, 100, 150, and 200 μm) while keeping the contact area same (50 × 50 μm^2^), no appreciable change in the resistance value was observed, both at 300 K as well as 333 K (Fig. 4d and Supplementary Fig. 17). Also, systematically varying the contact pad area (50 × 50 μm^2^, 100 × 100 μm^2^, 150 × 150 μm^2^ and 200 × 200 μm^2^) while keeping the channel length same (50 μm), the resistance values did not change noticeably, both at 300 and 333 K (Fig. 4e and Supplementary Fig. 18). To strengthen the IMLT effect, we have fabricated Cu(Cys)_2_ thin films of different thickness values (~750 nm, ~980 nm, and ~1.5 μm) and upon changing the temperature of the samples from 300 to 333 K, an increase in the conductance value by 10^10^ fold was consistently observed (Supplementary Figs. 19 and 20). Such robustness in the *I*–*V* profiles along with the reversibility of the IMLT phenomenon, further ruled out the possibility of diffusion of Au from the contact pads into the Cu(Cys)_2_ thin film. Therefore, keeping in mind the intricate role of the electronic structure of the interface, our various complementary electrical transport measurements highlighting the consistent linear *I*–*V* profiles within ±0.5 V, in both in-plane and cross-plane modes, endorsed IMLT phenomenon as a unique property of the Cu(Cys)_2_ thin film.

## Discussion

**Fig. 5:**
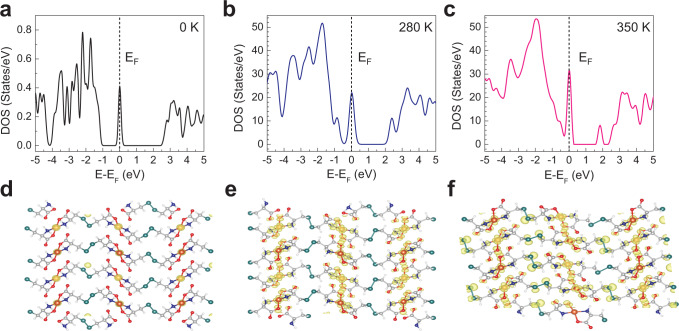
Unravelling mechanistic insights of the IMLT phenomenon. Total density of state (DOS) plots for the Cu(Cys)_2_ structure at 0 K (**a**), 280 K (**b**), and 350 K (**c**) (*E*_F_ is marked by the dotted black lines). Partial charge density plots for the Cu(Cys)_2_ structure around *E*_F_ (±0.5 eV) at 0 K (**d**), 280 K (**e**), and 350 K (**f**) where carbon, hydrogen, nitrogen, oxygen, sulfur, and copper atoms are represented by grey, white, blue, red, dark cyan and orange colors, respectively; and electron accumulation is represented by yellow isosurface. Source data are provided as a Source Data file.

In order to get mechanistic insights into the origin of such an electronically driven IMLT phenomenon, DFT calculations combined with molecular dynamics (MD) simulations were performed on the Cu(Cys)_2_ system at three different temperatures (0, 280, and 350 K). The total density of states (DOS) plots obtained at 0, 280, and 350 K are presented in Fig. 5. An important observation in the DOS plot at 0 K was the appearance of a discrete state around Fermi energy (*E*_F_, ±0.25 eV) which was mainly originating from the Cu atoms, as identified in the atom-projected partial density of states (PDOS) plot (Supplementary Fig. 21). Also, the discrete state was populated by approximately one electron density, residing mostly on the *t*_2*g*_ set of the 3*d* orbitals of Cu (Supplementary Fig. 22); and the energy gap between the valence band (VB) minimum and the discrete state was found to be ~1.0 eV. Therefore, Cu(Cys)_2_ can be ascribed as a Mott-type insulator^30,52^. Upon increasing the temperature from 0 to 280 K, the width of the discrete state around *E*_F_ was increased (±0.5 eV), and with a significant contribution from S atoms (Supplementary Fig. 21), the VB minimum shifted towards *E*_F_, thereby reducing the energy gap. Remarkably, at 350 K, the discrete state at *E*_F_ was merged with the VB and a metal-like continuous density of states was realized viz. overlapping of the Cu-based states via the S-based state (Supplementary Fig. 21). Additionally, total charge density plots (Supplementary Fig. 23) and partial charge density plots (Fig. 5) were carefully examined and Bader charge analysis^39,46,53^ was carried out to understand the distribution of charge on various atoms at different temperatures which showed that S atom released and Cu atom accumulated charges at higher temperatures (Supplementary Table 1) so that the respective electronic band structures were affected by the overall charge re-distribution across the high-symmetry points (Supplementary Fig. 24). Thus, IMLT in our Cu(Cys)_2_ system can be attributed to be due to the onset of an electronic coupling primarily involving 3*d* and 3*p* orbitals of Cu and S atoms, respectively (possibly, via 2*p* orbitals of N atoms)—without any appreciable change in the structure and/or coordination. Notably, earlier studies revealed that infinite chains and planes of (-M-S-)_∝_ in MOF structures (so-called “through-bond” approach)^24,54,55^, specifically the (-Cu-S-)_∝_ linkage, could enable efficient charge-transport leading to high-electrical conductivity in the systems. The present study is clearly unraveling the innovative ‘through-space’ avenue (-Cu----S-)_∝_ as well as stimulates the usage of Mott-type insulator Cu(Cys)_2_ in neuromorphic device applications^52^. Finally, we have theoretically calculated electrical conductivity values for the Cu(Cys)_2_ system at different temperatures, which qualitatively complemented our experimental observation on the IMLT phenomenon, specifically the trend, and certainly encourage further investigations (Supplementary Fig. 25 and Table 2).

In summary, we have presented a thermally driven and fully reversible IMT-like transition in a thin film of Cu(Cys)_2_ at ambient pressure and a little above room temperature. The transition was achieved without a noticeable change in the structure and assigned to be of mainly electronic origin, viz., a charge cross-over phenomenon. Successful capturing of the IMLT in the photo-lithographically patterned sample, along with the robust current-voltage characteristics, ensured the device application possibilities of the Cu(Cys)_2_ thin film. This work is anticipated to provide a new platform for studying the coupling of charge, spin, and lattice degrees of freedom in correlated biological reticular materials. Thermally-driven supercurrent in biological MOFs is also envisioned.

## Methods

### Chemicals

Copper acetate [Cu(OAc)_2_.H_2_O], L-Cysteine, 11-Merceptoundecanoic acid (MUDA), and Au(111) substrate (100 nm Au coated Si wafer) were purchased from Sigma-Aldrich and ethanol from Merck and used without any further purification except Au(111) substrate.

### Functionalization of Au substrate

Au substrate was cleaned by dipping into Piranha solution (H_2_SO_4_ (95–98%)/H_2_O_2_ (30%); v/v 3:1) for 30 min (note: proper safety care must be taken) and washed with Milli-Q water followed by ethanol and then dried under the stream of N_2_ gas. Subsequently, the Au substrate was functionalized with a carboxy-terminated (-COOH) self-assembled monolayer (SAM) by dipping into the 1 mM MUDA solution in ethanol/acetic acid (v/v 9:1) for 48 h followed by washing with ethanol and dried with N_2_ gas^56,57^.

### Fabrication of Cu(Cys)_2_ thin film

MUDA functionalized Au substrate was immersed in 1 mM ethanolic solution of Cu(OAc)_2_.H_2_O kept at 330 K for 30 min, followed by washing with ethanol and subsequently in 2 mM Cysteine solution in ethanol–water (1:1) mixture kept at 330 K for 30 min (washed with ethanol in between followed by drying with a stream of N_2_ gas) to complete one LbL cycle^50^. Thirty cycles of LbL were performed to get uniform Cu(Cys)_2_ thin film (thickness values were estimated to be ~1.2 μm). Employing the same experimental conditions, thin films of Cu(Cys)_2_ with thickness values of ~750 nm, ~980 nm, and ~1.5 μm were also fabricated, corresponding to LbL growth of 20 cycles, 25 cycles, and 35 cycles, respectively.

### Synthesis of Cu(Cys)_2_ powder

Cu(OAc)_2_·H_2_O (1 mM) and Cysteine (2 mM) solutions were prepared in ethanol and ethanol–water (1:1) mixture, respectively, and mixed together in a round bottom flask. The solution was kept at 330 K with constant stirring for the next 3 days. A sky-blue colored powder was collected after centrifugation with water and ethanol three times alternatively.

### Characterizations

The surface morphologies of thin film and bulk performed by Zeiss Ultra Plus FESEM. Contact angle measurements were measured using Holmarc’s Contact Angle Meter. Out-of-plane XRD data were recorded at room temperature and high temperature using a Bruker D8 Advance diffractometer using Cu *K*_α_ radiation (*λ* = 1.5406 Å). Raman spectra (*λ*_exc_ = 632.8 nm) were recorded at Raman microscope (LabRAM HR, HoribaJobinYvon) with a 50× objective lens, and the spectral resolution of the system is ~1 cm^−1^. FTIR spectra were collected in the range of 400–4000 cm^−1^ with a resolution of ~4 cm^−1^ using KBr pellets (NICOLET 6700 spectrophotometer). High-resolution XPS spectra were recorded by using a Thermo Fisher Scientific ESCALAB Xi+ for both bulk and thin film, and a hand-book^58^ was referred for the assignments of various photoemission signals. ESR was recorded using JES - FA200 ESR Spectrometer with an X band on the bulk sample. Gas adsorption and desorption isotherms were collected at 77 K for N_2_ gas on the BelSorpmax instrument (pore size distribution was obtained from the standard H–K model). HRTEM images were recorded on drop casted samples (methanolic dispersion) over a 200 mesh Cu grid using JEOL USA JEM-2200 FS Transmission Electron Microscope (scratched sample in case of thin film). TGA profiles were recorded using Perkin-Elmer thermal analyzer STA 6000 model. Electrical transport measurements (current–voltage, *I*–*V* profiles) on various thin film samples were carried out using a Keithley 4200 SCS Parameter Analyser system attached to an Everbeing probe station with eutectic gallium indium (EGaIn) alloy, tungsten tip, Au and Pt pads and direct Au tip as the contact electrodes, in two-probe configuration. During the electrical transport measurements at ambient pressure, relative humidity (RH) values at 300 and 333 K were noted to be ~46% and ~38%, respectively.

### Photolithography

It was performed by LW405 Laser writer, followed by the deposition of Au by using LTE sputter 080. A standardized procedure^49^ was adopted as follows: Step-1: Spin coating of photoresist (S1813; ~0.2 to ~0.3 ml; thickness ~2 μm) on the Cu(Cys)_2_ thin film; Step-2: a patterned mask was applied to block the UV light, and only unmasked regions of the material were exposed to the UV light; Step-3: the exposed photo-sensitive material is degraded by the UV light; Step-4: the degraded material is dissolved in developer (MF319) solution; Step-5: Au (~100 nm) was sputtered on the patterned thin film; and Step-6: patterned thin film was sonicated in acetone for 1–2 min to dissolve the photoresist and resulted into the well-defined Au pads on the Cu(Cys)_2_ thin film.

### Computational studies

The unit cell of the Cu(Cys)_2_ system was modeled by considering experimentally obtained data on the Zn(Cys)_2_ system^44^ (CCDC 971360), where Zn metal ions were replaced with Cu ions using an open-source molecular builder and visualization tool Avogadro^59^. The geometrical optimizations and the extraction of electronic structure information were achieved by using the Vienna Ab-initio Simulation Package (VASP) with projector augmented wave method^60^. The Perdew–Burke–Ernzerhof functional of generalized gradient approximation^61^ was employed as exchange-correlation functions to estimate the accurate DOS and band structure^62^ of the Cu(Cys)_2_ system. The conjugate gradient method was used to obtain the energy-optimized structure, and the optimization process was carried out until the interatomic forces in the system were reduced to 0.001 eV/Å. The Brillouin zone integration is performed using a 5 × 5 × 3, Monkhorst–Pack grid. To study the temperature-dependent electronic structure and calculate the electronic conductivity, MD simulations were carried out at different temperatures using Parrinello and Rahman method as implemented in VASP. In our simulation process, we have used Langevin dynamics^63–65^ for the isobaric–isothermal ensemble where the Brillouin zone was sampled at the Gamma point 1 × 1 × 1 and a PAW basis-set with energy cut-off of 600 eV was considered. To equilibrate the sample at a particular temperature, we used 1.0 fs per step and simulated the sample for a minimum of 150,000 MD steps. Simulated mean temperatures achieved at thermal equilibrium were 279.16 K and 350.61 K for 280 K and 350 K, respectively. The thermally equilibrated samples were further used to study the electrical conductivity of the system at different temperatures. Conductivity calculations were performed by using the Boltzmann transport method implemented in BOLTZWANN code^66^ distributed with Wannier90 package^67^. In this method, the semi-classical Boltzmann transport equations were solved for the periodic infinite system under the constant relaxation-time approximation, where the band energies and band derivatives were obtained via Wannier interpolations. We have used plane wave (PW) results obtained from VASP to construct the maximally localized Wannier functions, which were interpolated accurately to calculate transport quantities on a coarse k-point reciprocal space grid. In the present calculation, highly dense 40 × 40 × 40 k-mesh was used for the interpolation in constructing tight-binding Hamiltonian matrix elements. All individual snapshots at different temperatures obtained from MD simulations were considered as the initial configuration. And maximally localized Wannier functions were derived by choosing the individual projection. Electrical conductivity was calculated for a chemical potential equal to 0 eV by minimizing the real space spread of individual Wannier functions. Bader charge analysis^53^ was performed using Henkelman’s Group program^68–70^.

### Crystal structure analysis

Structural characterization of the thin film was carried out by performing the Rietveld analysis of the XRD data using the FullProf program^45^. Our analysis shows that crystallites are preferentially oriented toward three directions which makes a real refinement using traditional software packages such as FullProf and GSAS difficult because, in these packages, it is onerous to include more than one preferred orientation direction. The initial crystal structural parameters for the refinement were taken from the Zn(Cys)_2_ system (CCDC 971360)^44^, with Zn(II) ions replaced by Cu(II) ions and energy-optimized by DFT calculations. In the present case, the diffraction pattern was simulated by considering the three preferred orientations (110), (−101), (01-1) with lattice parameters, *a* = 19.999(3) Å, *b* = 9.434(2) Å, *c* = 5.484(1) Å, *α* = *β* = *γ* = 90°. To account for the spectrum shifting, variation along the longer *a*-axis was expected due to the strain in thin films or the existence of a defect state during the initial cycles of the LbL growth at solid–liquid interface. Therefore, contraction along *a*-axis (*a* = 18.980 Å) was considered during the simulation along with the aforementioned cell parameters, with preferred orientation along (100), with the maximum fraction of 5%, which led to the good match of refined diffraction pattern to the experimental diffraction pattern.

### Reporting summary

Further information on research design is available in the Nature Portfolio Reporting Summary linked to this article.

## Supplementary information

Supplementary Information

Reporting Summary

## Data Availability

All data supporting the findings of this study are available within this article and its Supplementary Information, as well as from the corresponding author upon request. Source data are provided in this paper.

## Source data

Source Data
