# Supplementary material for: Insulator-to-metal-like transition in thin films of a biological metal-organic framework
Source: Nat Commun. 2023 May 19;14:2857. doi: 10.1038/s41467-023-38434-4 (PMC10198987; doi:10.1038/s41467-023-38434-4)
Supplement: Supplementary file 1 — Supplementary Information [file 41467_2023_38434_MOESM1_ESM.pdf]

Supplementary information for

Insulator-to-metal-like transition in thin films of a biological metal-organic framework

Pooja Sindhu[1], K. S. Ananthram[2], Anil Jain[3,4], Kartick Tarafder[2] & Nirmalya Ballav[1*]

[1]Department of Chemistry, Indian Institute of Science Education and Research, Dr. Homi Bhabha Road, Pune – 411 008, India

[2]Department of Physics, National Institute of Technology Karnataka, Surathkal, Mangalore – 575 025, India.

[3]Solid State Physics Division, Bhabha Atomic Research Centre, Mumbai – 400085, India

[4]Homi Bhabha National Institute, Anushakti Nagar, Mumbai – 400094, India

*Corresponding author. Email: nballav@iiserpune.ac.in

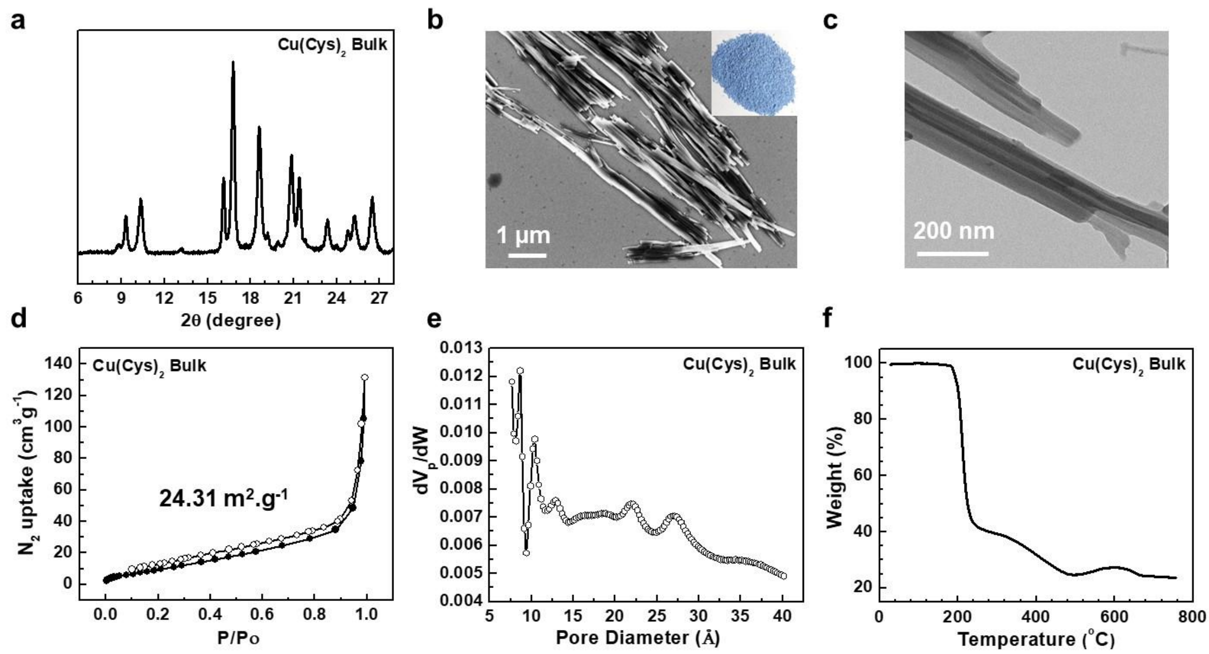

Supplementary Fig. 1. (a) Powder-XRD pattern revealing crystalline nature of the bulk Cu(Cys)$_2$. (b) FESEM image (inset: optical image of the powder) and (c) TEM image indicating the rod-like morphology of the Cu(Cys)$_2$ crystallites. (d) N_2 gas sorption profile along with the estimated BET surface area and (e) pore diameter profile obtained from standard H-K model showing its porous nature. (f) TGA profile depicting the thermal stability of the Cu(Cys)$_2$ bulk. Source data are provided as a Source Data file.

Trials on Single-Crystal Growth: To achieve the single crystal of Cu(Cys)$_2$, different methods have been implemented by symmetrically varying various parameters in the (1) vapour diffusion method, (2) liquid-liquid diffusion/layering method, and (3) solvothermal method, as described below:

1. Vapour Diffusion Method: (i) A mixture of copper acetate and cysteine (1:2) in ethanol/water mixture (1:1) was placed in the inner vial and the more volatile solvent was placed into the outer vial and allowed the reaction at room temperature for next 3 days. Outer vail solvents were varied such as ethanol, methanol, acetone and diethyl ether. (ii) Similar reactions were carried out at 330 K.

2. Liquid-Liquid Diffusion Method: (i) Cysteine (10 mM) was dissolved in ethanol/water mixture (1:1) and the solvent of copper acetate (5 mM) was varied such as DMF, DMSO, THF, NMP, Acetonitrile and ethyl acetate. (ii) Similar reactions were carried out with cysteine (10 mM) solution in water.

3. Solvothermal Method: (A) 4 ml mixture of Cu(OAC)$_2$ (5 mM in ethanol) and cysteine (10 mM in ethanol-water (1:1) mixture) sealed in Teflon vessel with stainless steel jacket was heated at 100ºC with the heating rate of 0.83ºC/min and kept at 100ºC for next 8 hours and slowly cool down to room temperature in 48 hours. The reaction was carried out in neutral, basic (by varying the NaOH addition) and acidic (by varying the HCl addition) conditions. Similar reactions were carried out by changing: (i) metal salt to Cu(NO$_3$)$_2$, (ii) metal solution in ethanol to DMF solvent, (iii) cysteine solution in water only, (iv) cysteine solution in water: solvent (DMF/ethanol) mixture, (v) NaOH base to KOH along with varied concentration, (vi) similarly, HCl to HNO_3 along with varied concentration, and (vii) HCl to acetic acid along with varied concentration. (B) Also, by changing the heating rate, reaction time and cooling time to RT.

In all above-mentioned conditions, no single crystal was observed, only the solid bulk or clear solution was achieved. Please note that the single crystal of Zn(Cys)$_2$ (Ref. 44) was also not obtained similar to our case unlike other amino acid MOFs.

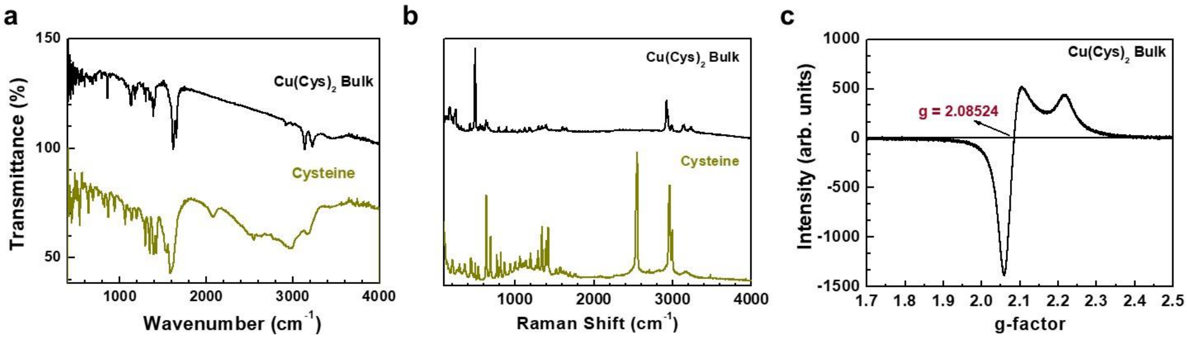

Supplementary Fig. 2. FTIR (a) and Raman (b) spectra of cysteine and Cu(Cys)$_2$ bulk. (c) EPR spectrum of the Cu(Cys)$_2$ bulk indicating the presence of Cu(II) (spin-1/2 system with g = 2.08) ion in the system. Source data are provided as a Source Data file.

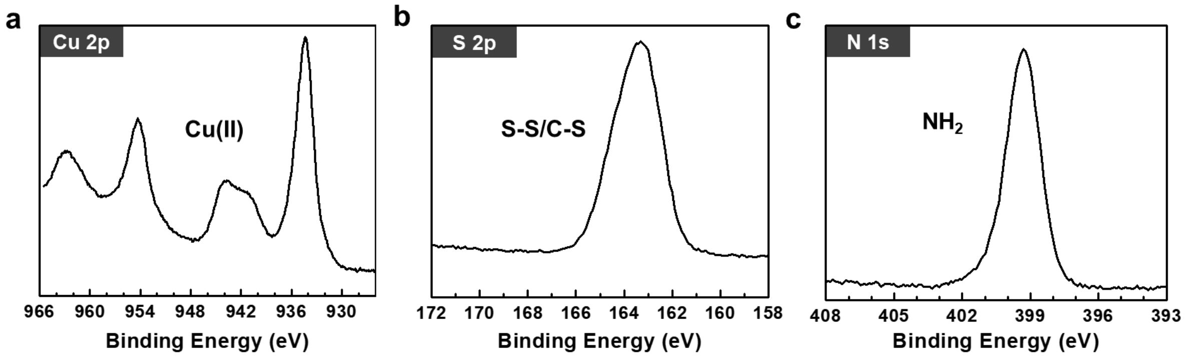

Supplementary Fig. 3. Cu2*p* (a), S2*p* (b) and N1*s* (c) XPS spectra of the $Cu(Cys)_2$ bulk confirming the presence of Cu(II) (Cu2$p_{3/2}$ peak at ~934.0 eV along with characteristic satellite features), S-S and C-S bonds (S2$p_{3/2}$ peak at 163.0 eV and no Cu-S bond for which S2$p_{3/2}$ peak would appear at ~162.0 eV) and NH_2 moiety (N1*s* peak at ~399 eV which is characteristic of amine). Source data are provided as a Source Data file.

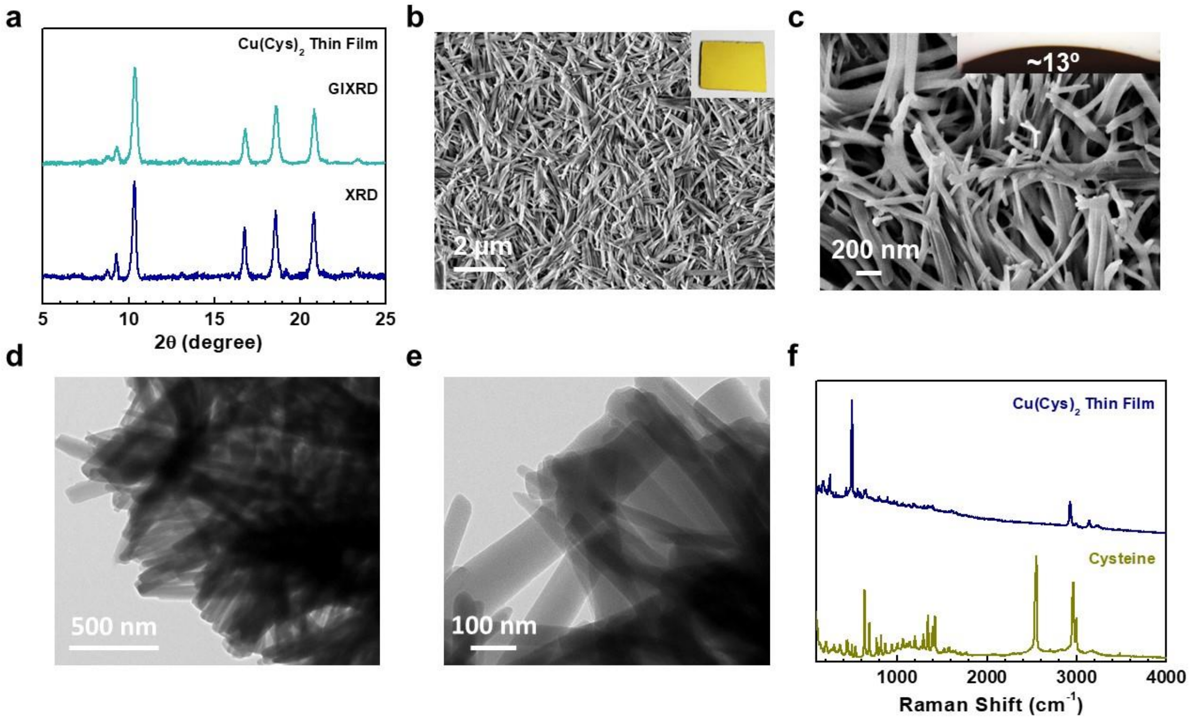

Supplementary Fig. 4. (a) GIXRD (grazing-incidence X-ray diffraction) and XRD patterns revealing crystalline nature of the $Cu(Cys)_2$ thin film. (b,c) FESEM image (inset of (b): Optical image of the $Cu(Cys)_2$ thin film and inset of (c): water contact angle) and (d,e) TEM image clearly indicating the rod-like morphology similar to the $Cu(Cys)_2$ bulk. (f) Raman spectra of the $Cu(Cys)_2$ thin film and the cysteine ligand. Source data are provided as a Source Data file.

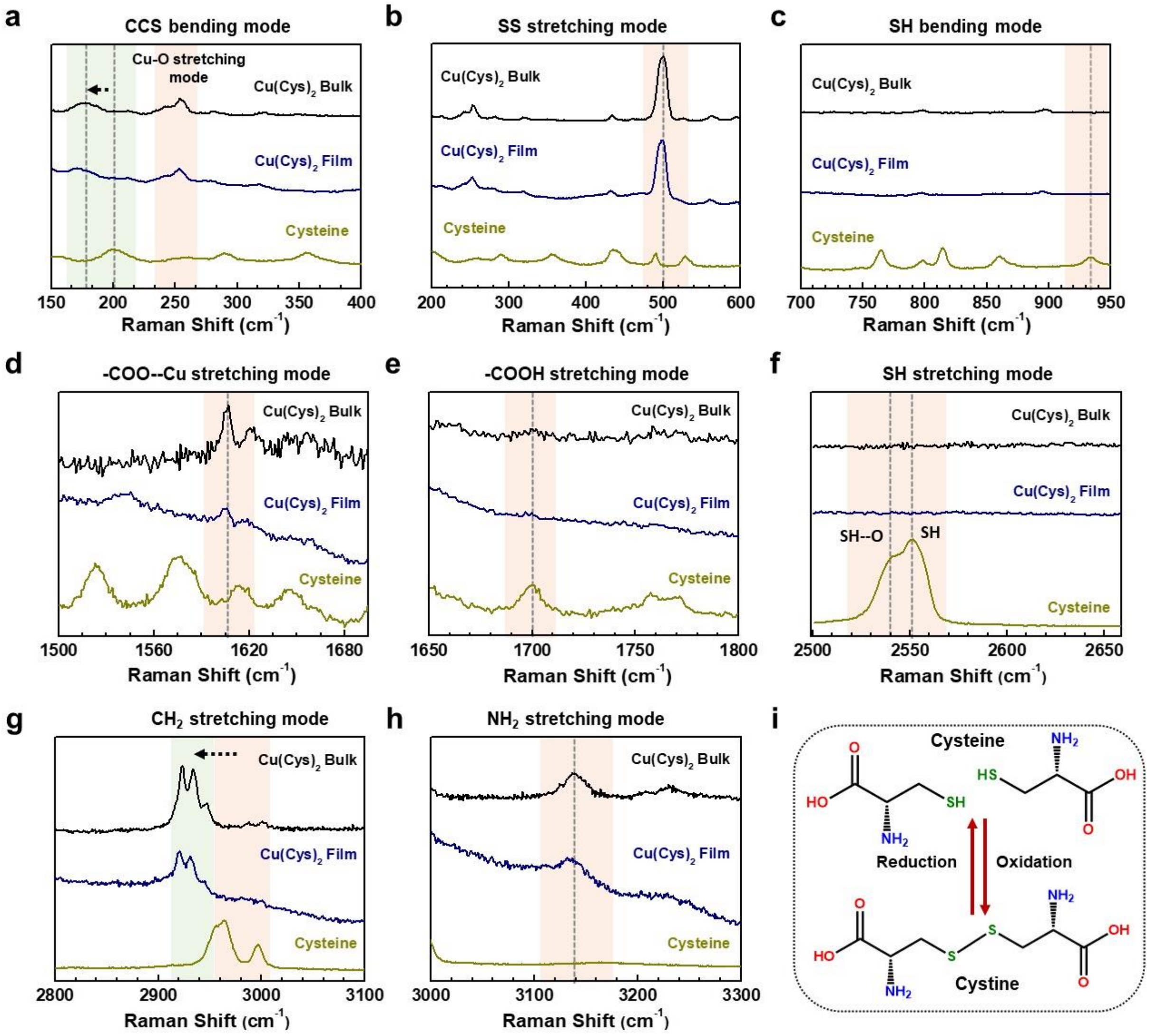

Supplementary Fig. 5. (a-h) Raman spectra of Cysteine (dark yellow), $Cu(Cys)_2$ thin film (blue) and $Cu(Cys)_2$ bulk (black). (i) Schematic representation of the conversion of cysteine to cystine. Our thorough analysis of the Raman spectra combined with the information from XPS data and other complementary characterizations confirmed the presence of $Cu(Cys)_2$ in bulk as well as in thin film. Source data are provided as a Source Data file.

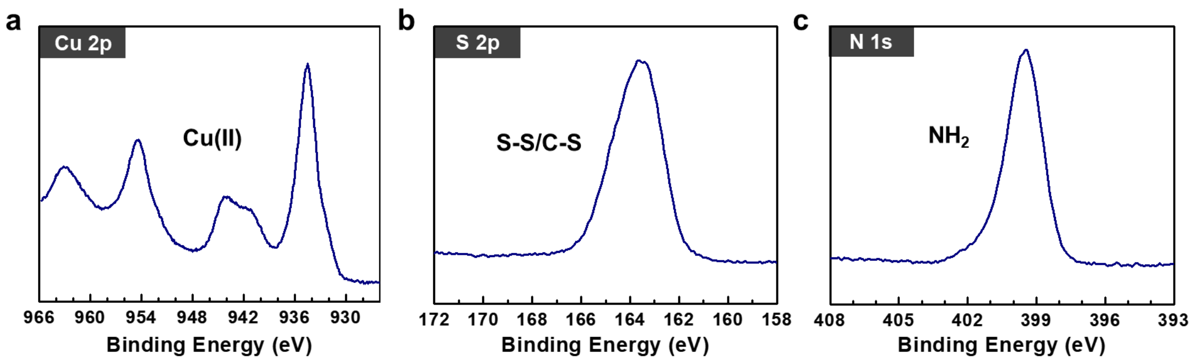

Supplementary Fig. 6. Cu2*p* (a), S2*p* (b) and N1*s* (c) XPS spectra of the $Cu(Cys)_2$ bulk confirming the presence of Cu(II) (Cu2$p_{3/2}$ peak at ~934.0 eV along with characteristic satellite features), S-S and C-S bonds (S2$p_{3/2}$ peak at 163.0 eV and no Cu-S bond for which S2$p_{3/2}$ peak would appear at ~162.0 eV) and NH_2 moiety (N1*s* peak at ~399 eV which is characteristic of amine). Source data are provided as a Source Data file.

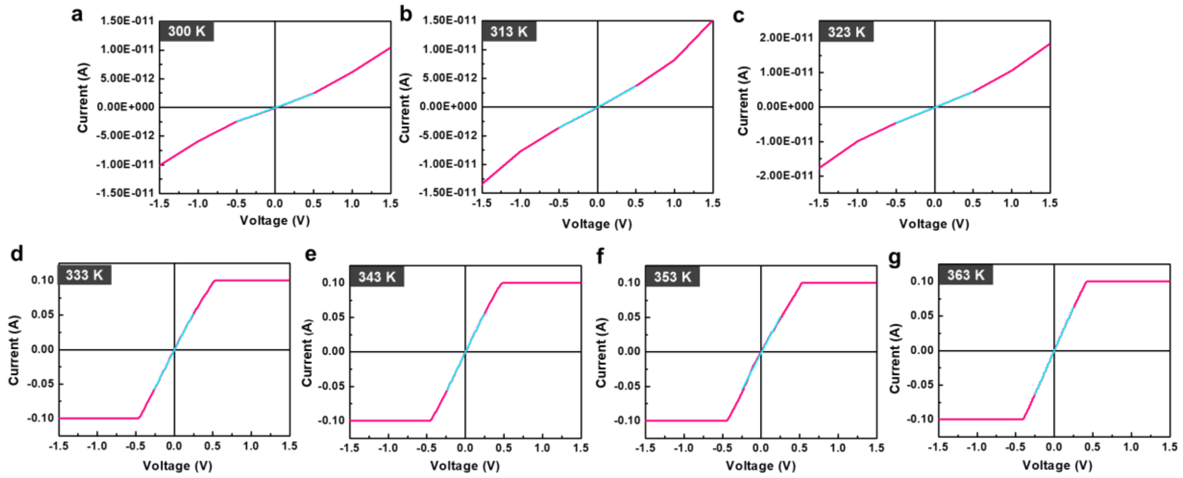

Supplementary Fig. 7. (a-g) Temperature-dependent *I-V* profiles of the $Cu(Cys)_2$ thin film in the cross-plane mode during heating from 300 K to 363 K using patterned Au pad (200x200 μm^2) as top electrode. Please note that the electrical conductance values are calculated by linear fitting (cyan line) at 300 K and 333 K from -0.5 V to +0.5 V and -0.25 V to +0.25 V respectively. Resistivity values calculated at 300 K and 333 K are 1.28 x 10^{10} Ωm and 10^{-1} Ωm, respectively. Source data are provided as a Source Data file.

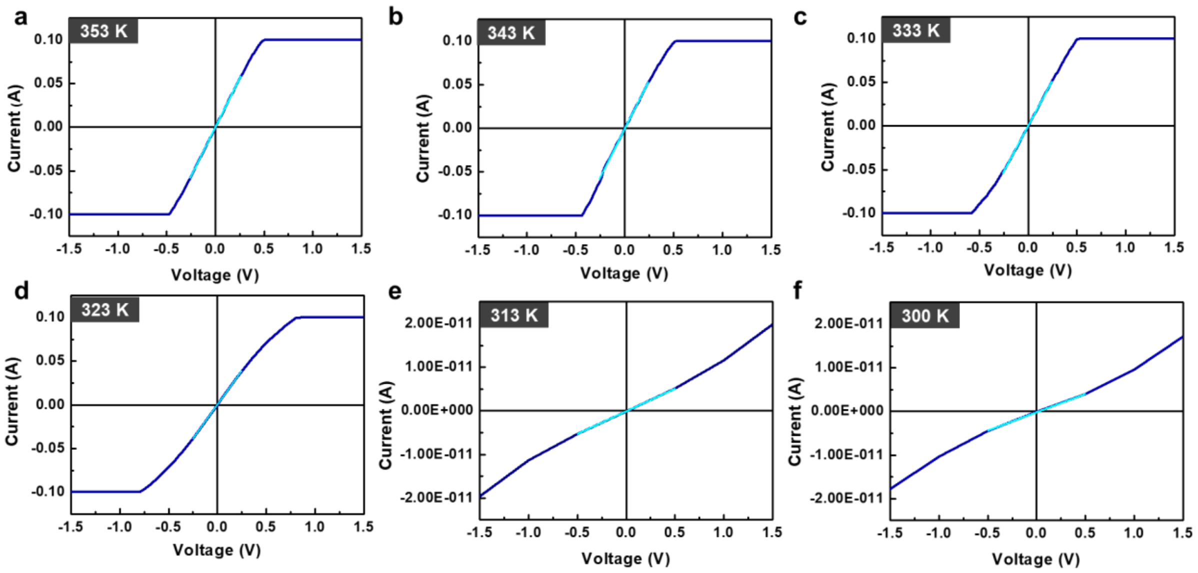

Supplementary Fig. 8. (a-f). Temperature-dependent *I-V* profiles of the $Cu(Cys)_2$ thin film in the cross-plane mode during cooling from 353 K to 300 K using patterned Au pad (200x200 μm^2) as top electrode. Please note that the electrical conductance values are calculated by linear fitting (cyan line) at 300 K and 333 K from -0.5 V to +0.5 V and -0.25 V to +0.25 V respectively. Source data are provided as a Source Data file.

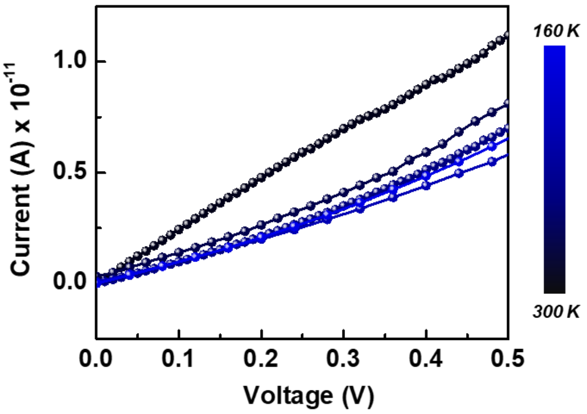

Supplementary Fig. 9. In-plane *I*-*V* profiles of the $Cu(Cys)_2$ thin film from room temperature (300 K) to low-temperature (160 K) indicating no appreciable change even by an order of magnitude. Low-temperature measurements were carried out by following the procedure recently standardized in our lab (Ref. 46). Source data are provided as a Source Data file.

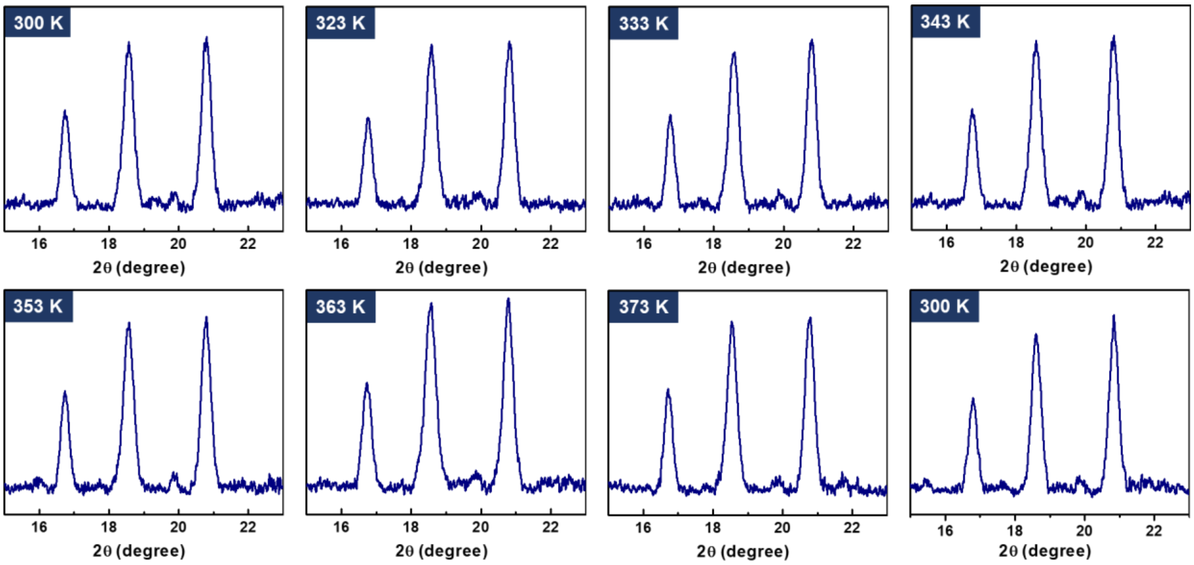

Supplementary Fig. 10. Temperature-dependent out-of-plane XRD patterns of the $Cu(Cys)_2$ thin film upon systematically varying the temperature from 300 K to 373 K (above the transition temperature) and back to 300 K. Apparently, no change in the pattern was observed. Source data are provided as a Source Data file.

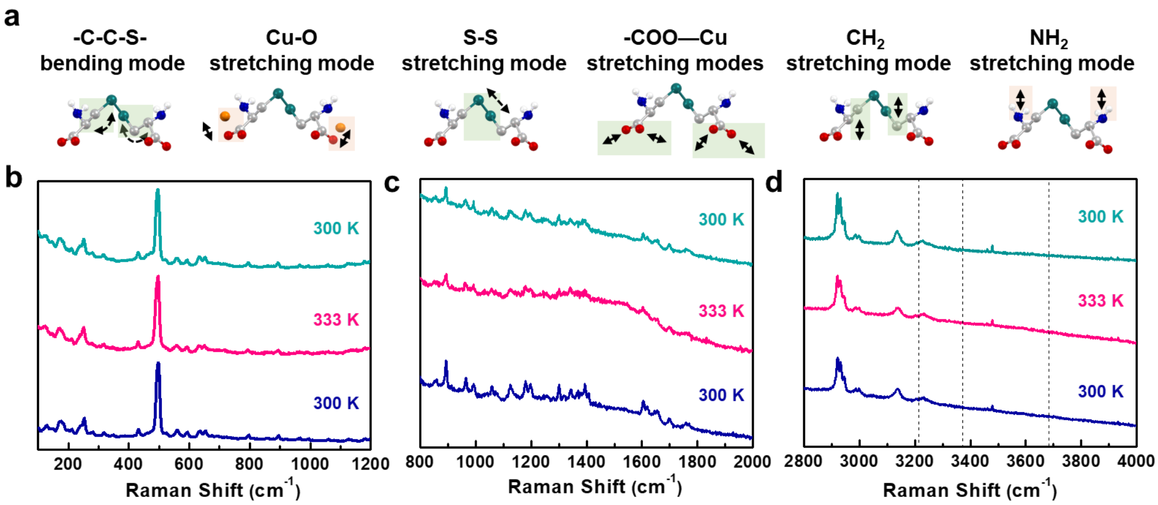

Supplementary Fig. 11. (a) Schematic representation of Raman spectral modes. (b-d) Raman spectra of the $Cu(Cys)_2$ thin film at various temperatures (300 K, 333 K and back to 300 K) indicating no change in the local structure and desorption-adsorption of water (characteristic vibrations are marked by dotted lines). Source data are provided as a Source Data file.

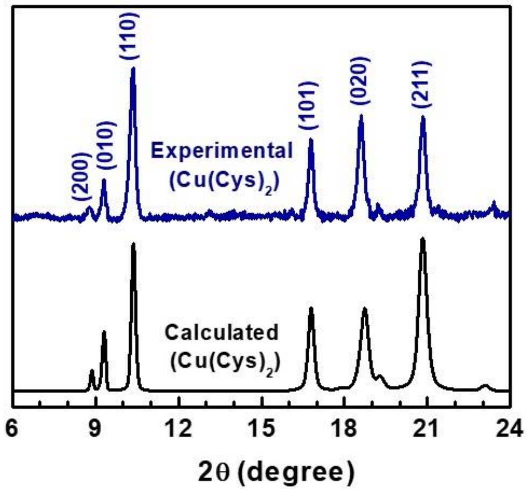

Supplementary Fig. 12. Experimental XRD pattern of $Cu(Cys)_2$ thin film and calculated XRD pattern from the Rietveld refinement. Diffraction peaks are also indexed. Source data are provided as a Source Data file.

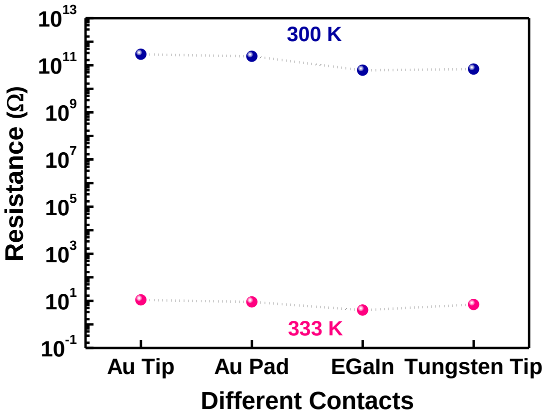

Supplementary Fig. 13. Plot of resistance of the $Cu(Cys)_2$ thin film with different contact electrodes, at 300 K and 333 K, consistently showing a change in the value by ~10^{10} fold upon changing the temperature from 300 K to 333 K. Source data are provided as a Source Data file.

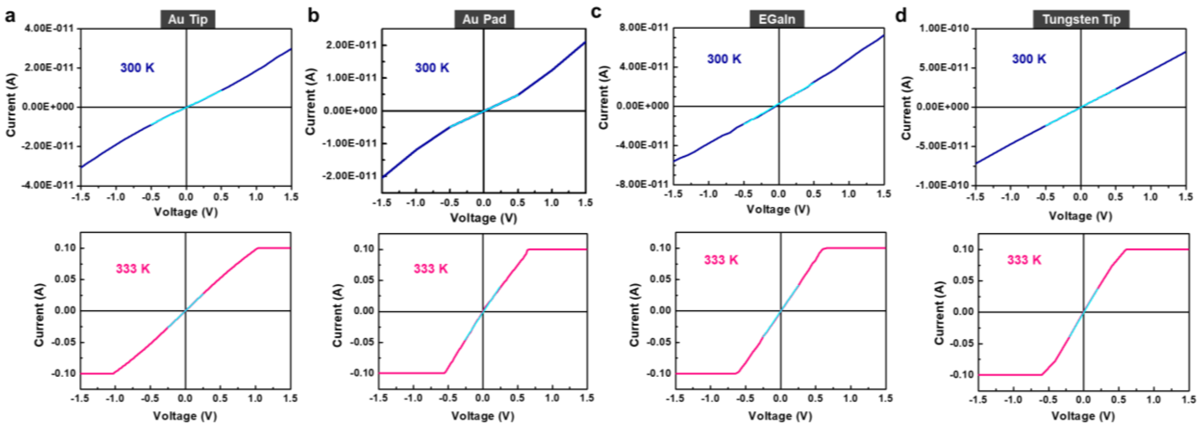

Supplementary Fig. 14. In-plane *I-V* profiles of the $Cu(Cys)_2$ thin film with different contact electrodes (a) Au tip (b) Au pad (c) EGaIn and (d) W tip, at 300 K and 333 K. Please note that the electrical conductance values are calculated by linear fitting (cyan line) at 300 K and 333 K from -0.5 V to +0.5 V and -0.25 V to +0.25 V respectively. Source data are provided as a Source Data file.

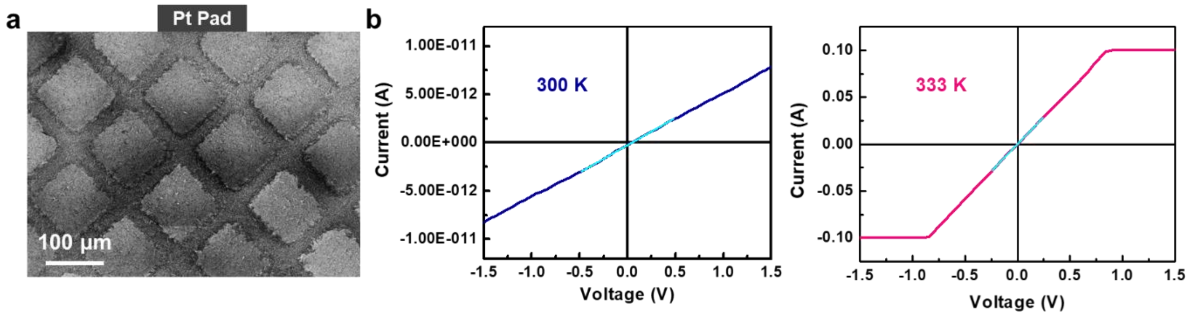

Supplementary Fig. 15. (a) FESEM image of Pt contact pad on the top of the $Cu(Cys)_2$ thin film (b) In-plane *I*-*V* profiles of the $Cu(Cys)_2$ thin film with Pt contact pads at 300 K and 333 K. Please note that the electrical conductance values are calculated by linear fitting (cyan line) at 300 K and 333 K from -0.5 V to +0.5 V and -0.25 V to +0.25 V respectively. Source data are provided as a Source Data file.

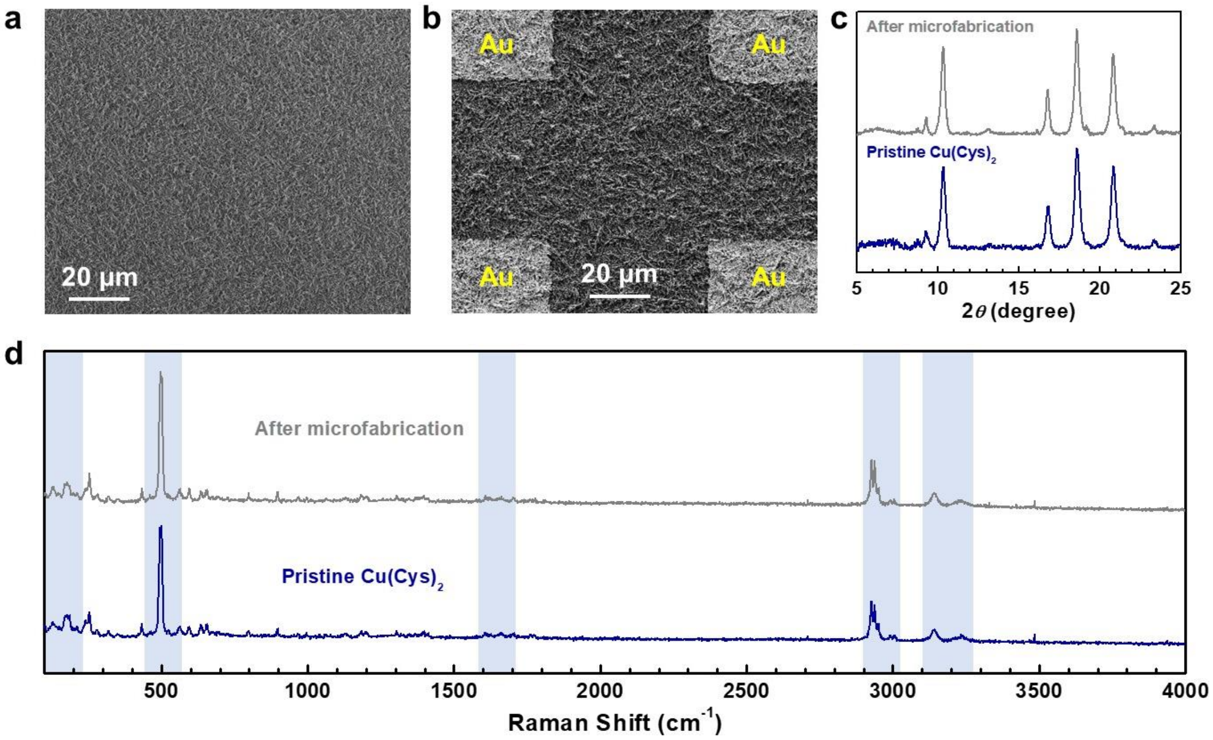

Supplementary Fig. 16. (a-b) FESEM images (c) XRD and (d) Raman spectra of the $Cu(Cys)_2$ thin film before and after microfabrication of the Au contact pads indicating no changes in the morphology as well as the structural integrity. Source data are provided as a Source Data file.

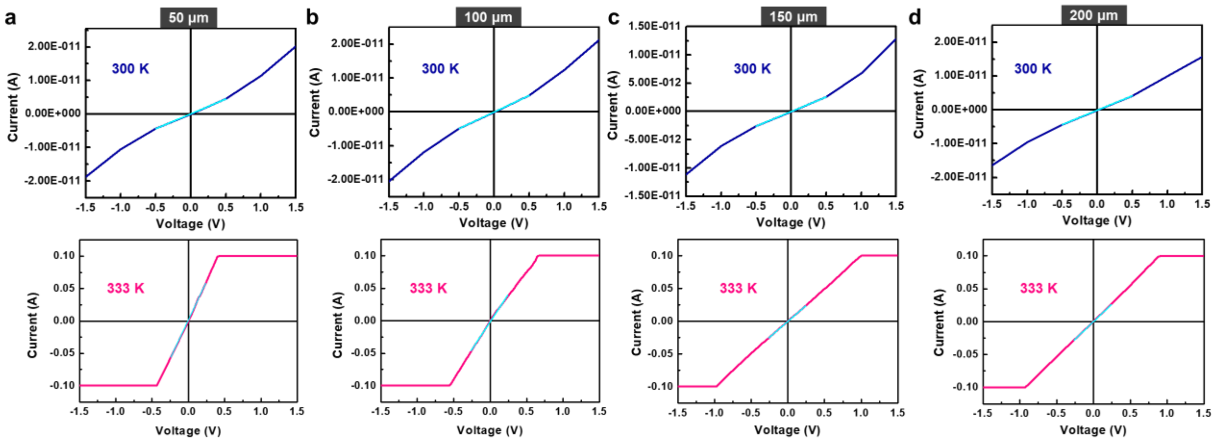

Supplementary Fig. 17. In-plane *I-V* profiles of the $Cu(Cys)_2$ thin film with the variation of channel length from 50 μm to 200 μm (a-d) at 300 K and 333 K. Please note that the electrical conductance values are calculated by linear fitting (cyan line) at 300 K and 333 K from -0.5 V to +0.5 V and -0.25 V to +0.25 V respectively. Source data are provided as a Source Data file.

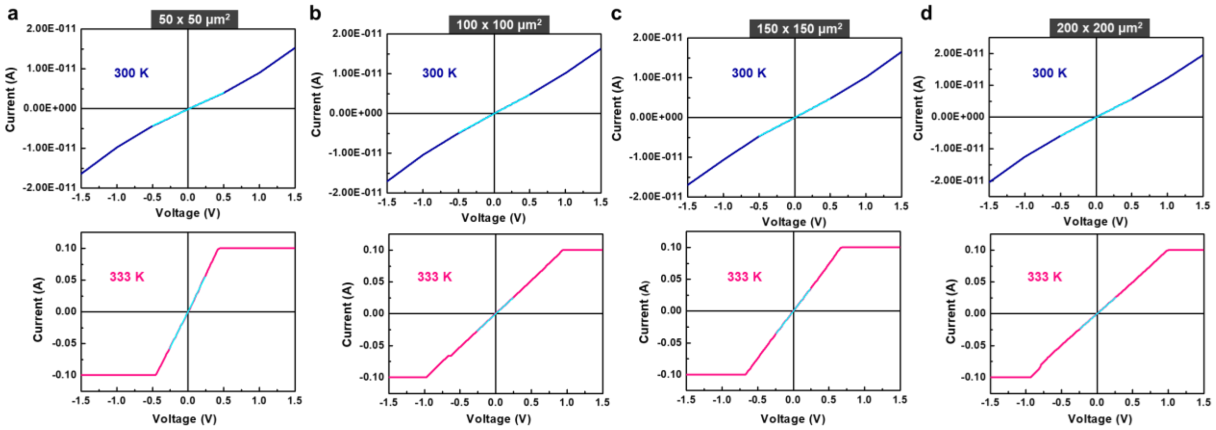

Supplementary Fig. 18. In-plane *I*-*V* profiles of the $Cu(Cys)_2$ thin film with the variation of contact area from 50 x 50 μm^2 to 200 x 200 μm^2 (a-d) at 300 K and 333 K. Please note that the electrical conductance values are calculated by linear fitting (cyan line) at 300 K and 333 K from -0.5 V to +0.5 V and -0.25 V to +0.25 V respectively. Source data are provided as a Source Data file.

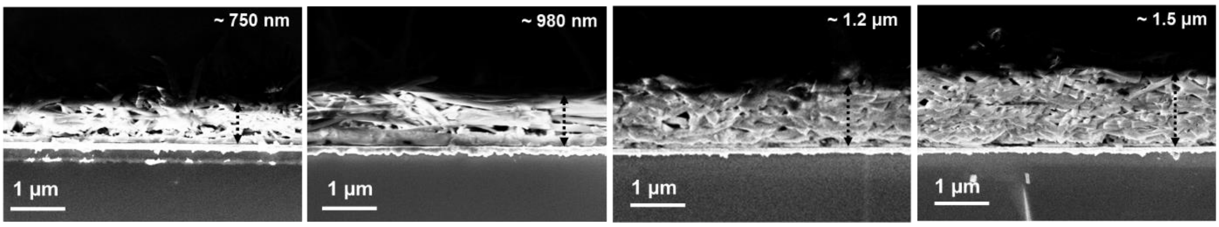

Supplementary Fig. 19. Cross-sectional FESEM images of the $Cu(Cys)_2$ thin films with different thickness values from ~750 nm to ~1.5 μm corresponding to LbL growth of 20 cycles, 25 cycles, 30 cycles and 35 cycles, respectively.

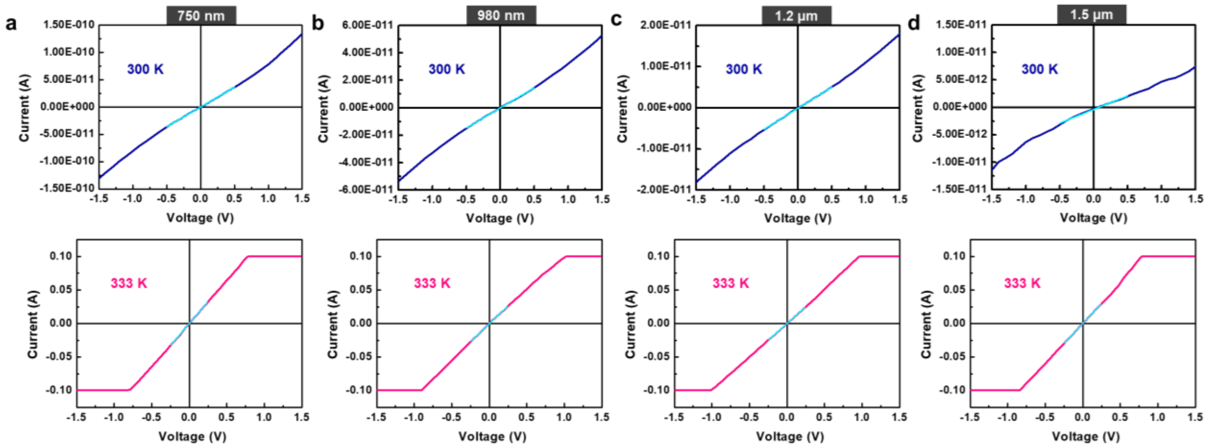

Supplementary Fig. 20. Current-Voltage (*I*-*V*) characteristic curves at 300 K (RT) and 333 K (HT) upon varying the thickness from 750 nm to 1.5 μm in $Cu(Cys)_2$ thin film. Please note that the electrical conductance values are calculated by linear fitting (cyan line) at 300 K and 333 K from -0.5 V to +0.5 V and -0.25 V to +0.25 V respectively. Source data are provided as a Source Data file.

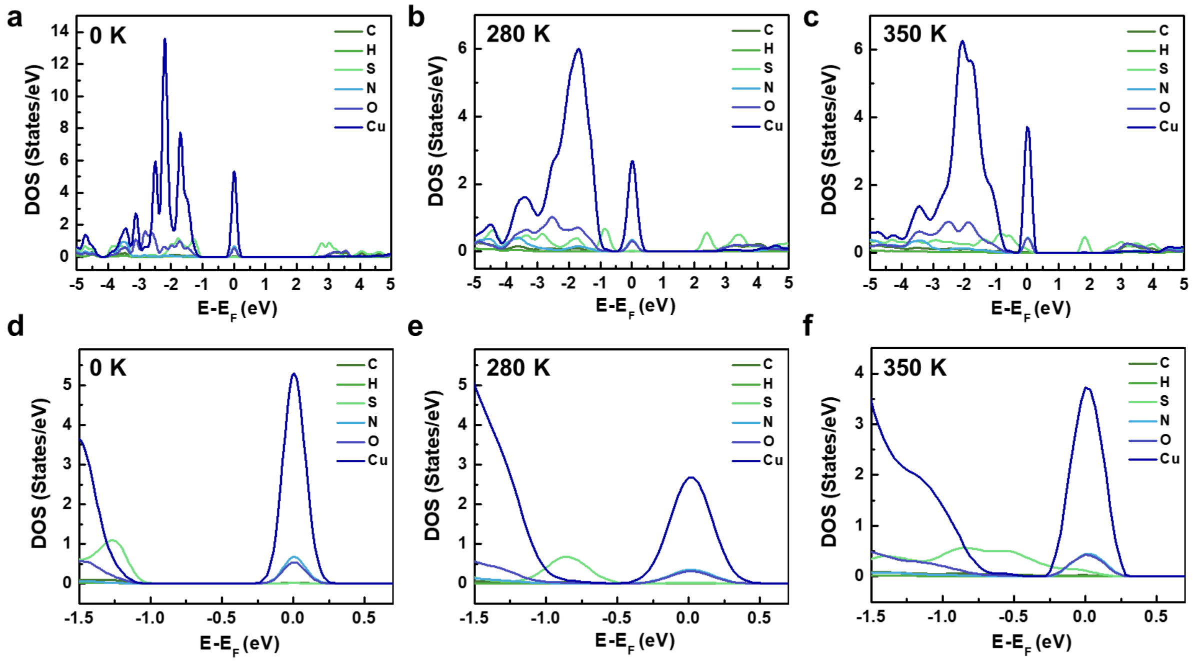

Supplementary Fig. 21. (a-c) Atom-projected partial density of states (PDOS) plots (top panels) at 0 K, 280 K and 350 K. (d-f) Respective zoomed-in sections around E_F are also presented (bottom panels). Source data are provided as a Source Data file.

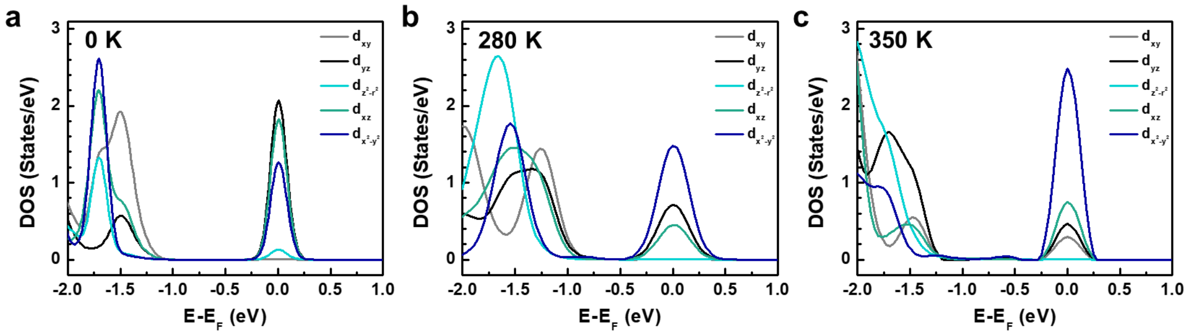

Supplementary Fig. 22. (a-c) 3d-orbitals contribution of Cu atom in the atom-project partial density of states at 0 K, 280 K and 350 K. Source data are provided as a Source Data file.

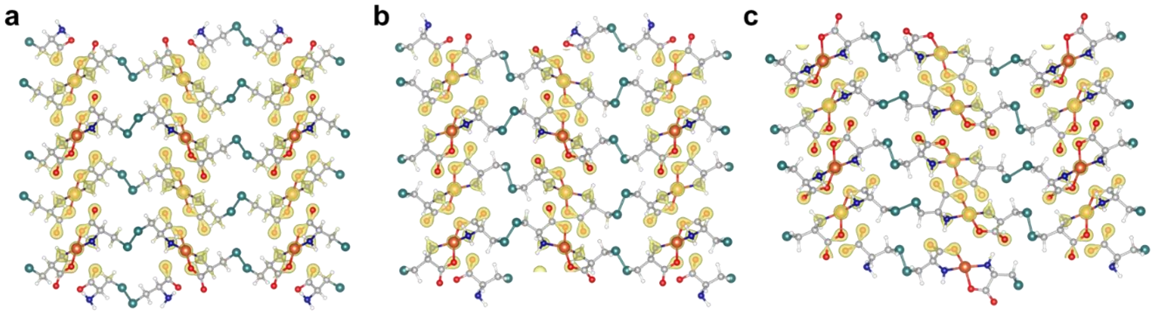

Supplementary Fig. 23. (a-c) Total charge density plots of $Cu(Cys)_2$ at 0 K (left panel), 280 K (middle panel) and 350 K (right panel) where carbon, hydrogen, nitrogen, oxygen, sulphur and copper atoms are represented by colors grey, white, blue, red, dark cyan and orange, respectively and electron accumulation is represented as yellow isosurface.

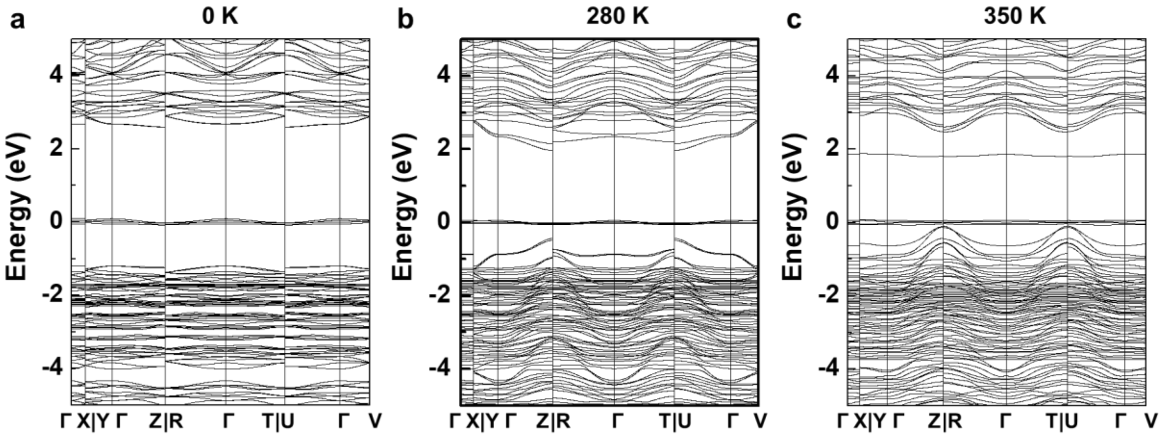

Supplementary Fig. 24. Band structures of the $Cu(Cys)_2$ system at (a-c) 0 K, 280 K and 350 K. Standard notations are used for the high-symmetry points (Ref. 62). Source data are provided as a Source Data file.

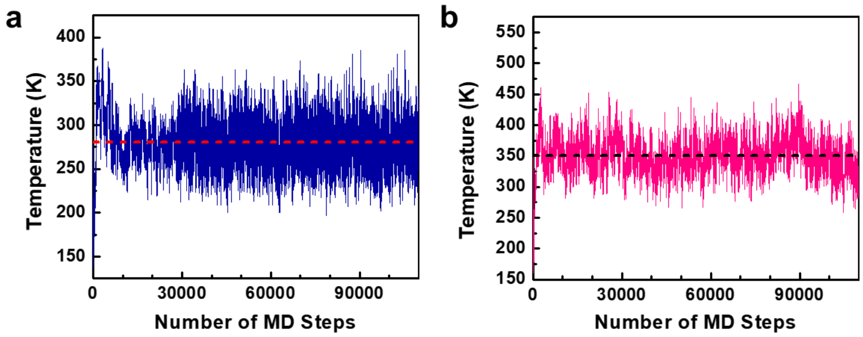

Supplementary Fig. 25. Temperature fluctuation during MD simulation at (a) 280 K and (b) 350 K. Source data are provided as a Source Data file.

Supplementary Table 1. Atoms-wise Bader charge contribution in the $Cu(Cys)_2$ structure at different temperatures.

Atoms (total number of atoms in the cell)	Effective Bader Charge (in electron)		
	**0 K**	**280 K**	**350 K**
**Cu (4 atoms)**	39.65	40.42	41.24
**S (8 atoms)**	46.95	46.19	45.22
**N (8 atoms)**	46.33	44.81	44.15
**O (16 atoms)**	111.71	109.04	109.48
**C (24 atoms)**	80.60	82.38	82.11
**H (40 atoms)**	38.74	41.13	41.77

Supplementary Table 2. Theoretical conductivity values from the Boltzmann transport method at various temperature. Temperature in MD simulations has a fluctuation of ± 20 K, hence the equilibrium temperature might not be the exact temperature that the system is mimicking. The deviation from the simulated temperature could be the reason why a higher conductivity value at 300 K (since it is close to experimental transition temperature of 333 K).

**Conductivity calculated in BOLTZWANN (in 1/Ohm/m)**	
**1 K**	764.74
**280 K**	10065.44
**350 K**	580924.35
